# Collagen-specific T-cell repertoire in blood and synovial fluid varies with disease activity in early rheumatoid arthritis

**DOI:** 10.1186/ar2553

**Published:** 2008-11-17

**Authors:** Francesco Ria, Romina Penitente, Maria De Santis, Chiara Nicolò, Gabriele Di Sante, Massimiliano Orsini, Dario Arzani, Andrea Fattorossi, Alessandra Battaglia, Gian Franco Ferraccioli

**Affiliations:** 1 Institute of General Pathology, Catholic University, Largo F Vito, Rome, 00168, Italy; 2 Department of Rheumatology, Catholic University, CIC, Via Moscati, Rome, 00168, Italy; 3 Institute of Hygiene and Biostatistics, Catholic University, Largo F Vito, Rome, 00168, Italy; 4 Department of Gynecology, Laboratory of Immunology, Catholic University, Largo F Vito, Rome, 00168, Italy

## Abstract

**Introduction:**

Type II collagen is a DR4/DR1 restricted target of self-reactive T cells that sustain rheumatoid arthritis. The aim of the present study was to analyze the T-cell receptor repertoire at the onset of and at different phases in rheumatoid arthritis.

**Methods:**

We used the CDR3 BV-BJ spectratyping to study the response to human collagen peptide 261–273 in 12 patients with DR4^+ ^rheumatoid arthritis (six at the onset of disease and six during the course of disease) and in five healthy DR4^+ ^relatives.

**Results:**

The collagen-specific T-cell repertoire is quite restricted at the onset of disease, involving approximately 10 rearrangements. Within the studied collagen-specific rearrangements, nearly 75% is shared among patients. Although the size of the repertoire used by control individuals is comparable to that of patients, it is characterized by different T-cell receptors. Part of the antigen-specific T-cell repertoire is spontaneously enriched in synovial fluid. The specific T-cell repertoire in the periphery was modulated by therapy and decreased with the remission of the disease. Failure of immunoscopy to detect this repertoire was not due to suppression of collagen-driven proliferation *in vitro *by CD4^+ ^CD25^+ ^T cells. Clinical relapse of the disease was associated with the appearance of the original collagen-specific T cells.

**Conclusions:**

The collagen-specific T-cell receptor repertoire in peripheral blood and synovial fluid is restricted to a limited number of rearrangements in rheumatoid arthritis. The majority of the repertoire is shared between patients with early rheumatoid arthritis and it is modulated by therapy.

## Introduction

Rheumatoid arthritis (RA) is an autoimmune chronic systemic inflammatory disease that affects mainly the joints, resulting in progressive functional impairment [1]. There is a general consensus that self-reactive mechanisms are largely responsible for the pathogenesis of RA. A large array of autoantibodies can be detected in the serum of RA patients, which strengthens the hypothesis that a loss of self-tolerance forms the basis of the disease [2-4]. The autoantibody response to a highly conserved protein, type II collagen, occurring during the first few years of the disease clearly indicates that self-reactive B cells are present [5-9]. On the other hand, the infiltration of T cells in the synovial tissue and the demonstration that there is autoreactivity of T cells against type II collagen [10-13] suggest that a cell-mediated immune response also plays a prominent role in joint inflammation. The T-cell mediated self-reactivity against type II collagen is strongly linked to human leucocyte antigen (HLA)-DR alleles DR4 and DR1 [14].

Type II collagen, a highly conserved sequestered antigen, has been proposed to be one of the targets of the self-reactive T cells that sustain RA. In experimental models, induction of collagen-specific responsiveness results in a disease similar to RA [15,16]. T cells that are specific to human collagen peptide 263–270 were observed to arise in several models of RA involving mice transgenic for human DR molecules [14,17].

Given this background, T cells specific for this epitope should be detectable in early RA [11]. However, clear and direct evidence of the presence of collagen-specific T cells in the joints of RA patients in the early phases of the disease has not been reported [11]. In this respect, third complementarity-determining region (CDR3)-β typing of T cells infiltrating the joints has been used, aiming to identify clones that are specifically enriched in the inflamed synovia. Some CDR3-β regions have been reported to accumulate in the joints during disease [18-20], and a study revealed that some of these were shared among several patients [21].

In the present study, we used the CDR3 BV-BJ (variable and joining beta chain) spectratyping to study the response to human collagen peptide 261–273 (huCollp261–273) in patients with early RA. We first identified T-cell receptor (TCR)-β rearrangements belonging to cells that proliferate in a peptide-dependent manner in the peripheral blood of one patient, at the onset of RA. The presence of the same TCR rearrangements was thereafter examined in five consecutive patients with early RA. We then looked for the enrichment of T cells with these TCRs in the synovial fluid at the same time point in the disease course. Finally, we monitored these specific TCRs in the peripheral blood during various phases of the disease during therapy.

We found that the huCollp261–273-specific TCR repertoire of the index patient at the onset of the disease was limited to few rearrangements, and part of this antigen-specific repertoire was spontaneously enriched in the synovial fluid of the patient during the acute phase of the disease. We found the majority of the repertoire to be shared among patients with early RA, whereas healthy control individuals exhibited a distinct set of public (shared among different individuals) TCRs. The presence of collagen-specific T cells in the peripheral blood was modulated by therapy, and remission of the disease was associated with a decrease in the collagen-specific TCR repertoire, whereas relapses of the disease were accompanied by reappearance of the same T-cell repertoire detected at the onset.

## Materials and methods

### Patients

The demographic and clinical characteristics of six patients with RA who were analyzed at the onset of the disease, six patients with longstanding RA and receiving treatment, and five healthy relatives (all DRB1 04 positive) are summarized in Tables 1 and 2. All of the patients satisfied the American College of Rheumatology criteria for RA [22]. We decided to study healthy relatives in order to identify DRβ1 matched healthy control individuals. Active disease or relapse were defined, respectively, by a Disease Activity Score (DAS) was above 3.7 or increased to above 3.7 [23]. The patients began treatment with methotrexate 20 mg/week; etanercept 25 mg twice a week was added after 3 months in order to achieve remission when high disease activity was still present. Patients and control individuals were characterized with respect to the HLA-DR haplotype by PCR using sequence-specific oligonucleotides, using the Inno-LiPA HLA-DRB1 Amp Plus kit (Innogenetics N.V., Ghent, Belgium), in accordance with the manufacturer's instructions. The test can yield ambiguous findings in some cases that result in more than one possible combination of HLA-DRB1 alleles. Patients were entered into our cohort only if all of the combinations included at least one DRB1 04 allele.

**Table 1:** Characteristics of six RA patients at the onset of disease

Characteristic	Details
Sex	All female
Age (years [mean ± SD])	47.7 ± 13.2
Disease duration (weeks [mean ± SD])	8.0 ± 0.2
Bone erosion (% of patients)	50%
RF IgM (% of patients)	66.7%
RF IgA (% of patients)	16.7%
Anti-CCP (% of patients)	83.3%
DAS (mean ± SD)	6.1 ± 0.6

Each of the patients satisfied the American College of Rheumatology criteria for RA. CCP, cyclic citrullinated peptide; DAS, Disease Activity Scale; RA, rheumatoid arthritis; RF, rheumatoid factor; SD, standard deviation.

**Table 2:** Characteristics of six RA patients with longstanding disease

Characteristic	Details
Sex	66.6% female
Age (years [mean ± SD])	56.8 ± 11.2
Disease duration (months [mean ± SD])	6.4 ± 3.0
Bone erosion (% of patients)	100%
RF IgM (% of patients)	50%
RF IgA (% of patients)	50%
Anti-CCP (% of patients)	100%
Remission according to DAS (% of patients)	50%

Each of the patients satisfied the American College of Rheumatology criteria for RA. CCP, cyclic citrullinated peptide; DAS, Disease Activity Scale; RA, rheumatoid arthritis; RF, rheumatoid factor; SD, standard deviation.

Informed written consent was obtained from all patients. The research is in compliance with the Helsinki Declaration. The research was approved by the local ethics committee.

### Index case

Patient OE, a 50-year-old woman, had symmetrical involvement of the large and small joints that had lasted for 12 weeks; she was positive for rheumatoid factor and anti-cyclic citrullinated peptide antibodies. No bone erosions were present on radiography. The patient satisfied the American College of Rheumatology criteria for RA [22]. Her DAS was 6.69. Her HLA haplotype was A31, A68; B35, B38; CW04, CW12; and DRB1*04, DRB1*11.

The patient gave her informed consent, allowing us to obtain blood and synovial fluid samples, and she received specific therapy and modification to her therapy over time. Samples for immunoscopy analysis (peripheral blood and synovial fluid) were collected before therapy. The patient started methotrexate 20 mg/week and methylprednisolone 0.25 mg/kg/day at 8 a.m. Because no improvement was observed, etanercept 25 mg twice a week was added after 3 months. After 3 more months the patient exhibited a good response and achieved partial remission (DAS > 1.6 and < 2.4) [23]. Therefore, methylprednisolone was stopped. The patient was maintained with methotrexate and etanecept thereafter, and although her conditions initially worsen (DAS increased to 5.03), at later time points she showed improvement. A second blood sample was drawn for immunoscopy analysis at week 60 after diagnosis (DAS 3.5). Clinical evaluation was performed 3 months later, and a third blood sample was drawn for immunoscopy analysis at this time point (DAS 3.2). Clinical evaluation was again performed 3 months later, and a fourth blood sample was drawn for immunoscopy analysis also at this time point (DAS 6). Clinical and serological data are reported in Table 1.

### huCollp261–273-specific T-cell proliferation

Peripheral blood mononuclear cells (PBMCs) were purified with Percoll gradient and seeded in 96-well plates (Costar Corp., Cambridge, MA, USA) at 5 × 10^5 ^cells/well in the presence of graded concentrations of human collagen peptides 250–264, 261–273 and 289–303. Culture medium was RPMI 1640 (Gibco BRL Life Technologies, Basel, Switzerland), supplemented with 2 mmol/l L-glutamine, 50 μmol/l 2-ME (mercaptoethanol), 50 μg/ml gentamicin (Sigma-Aldrich, St Louis, MO, USA), and 1% human AB serum. Seventy-two hours later, antigen-specific T-cell proliferation was assessed by [^3^H]-thymidine incorporation.

### TCR repertoire analysis

Repertoire analysis was performed using a protocol described previously [24] but with modification. Briefly, PBMCs were cultured in the presence or absence of 20 μg/ml peptide for 3 days in RPMI-1640 medium (Sigma-Aldrich), supplemented as described above. The effect of the 3 days of culture on apoptosis of T cells was measured in a preliminary experiment by labelling cultured cells with anti-CD3-PC5 and anti-annexin V-FITC monoclonal antibodies. The percentage of apoptotic cells, evaluated by FACScan flow cytometer (Becton Dickinson) as annexin V^+ ^CD3^+ ^cells, was 20.4% in the sample obtained after Percoll separation, 23.2% for cells cultured in the absence of peptide antigen, and 29.3% for cells cultured in the presence of the antigen. Total RNA was isolated from cell suspensions using RNeasy Mini Kit (Qiagen GmbH, Hilden, Germany), in accordance with the manufacturer's instruction. cDNA was synthesized using an oligo-dT primer (dT15; Gibco BRL Life Technologies). From each cDNA, PCR reactions were then performed. Sequences of BV-, Cβ- and BJ-specific primers were deduced from the IMGT (ImMunoGeneTics) database, following the nomenclature of Currier and coworkers [25], and are summarized in Table 3.

**Table 3:** BV-, Cβ- and BJ-specific primers

PRIMER	SEQUENCE
CB1A	GGGTGTGGGAGATCCTGC
BV1	CCGCACAACAGTTCCCTGACTTGC
BV3	CGCTTCTCCCTGATTCTGGAGTCC
BV4	TTCCCATCAGCCGCCCAAACCTAA
BV5	GATCAAAACGAGAGGACAGC
BV6A	GATCCAATTTCAGGTCATACTG
BV6B1	CAGGGCCAGAGTTTCTGAC
BV6B2	CAGGGCTCAGAGGTTCTGAC
BV7	CCTGAATGCCCCAACAGCTCT
BV8	GGTACAGACAGACCATGATGC
BV9	TTCCCTGGAGCTTGGTGACTCTGC
BV10	CCACGGAGTCAGGGGACACAGCAC
BV11	GTCAACAGTCTCCAGAATAAGG
BV12	TCCYCCTCACTCTGGAGTC
BV13A	GGTATCGACAAGACCCAGGCA
BV13B	AGGCTCATCCATTATTCAAATAC
BV14	GGGCTGGGCTTAAGGCAGATCTAC
BV15	CAGGCACAGGCTAAATTCTCCCTG
BV16	GCCTGCAGAACTGGAGGATTCTGG
BV17	TCCTCTCACTGTGACATCGGCCCA
BV18	CTGCTGAATTTCCCAAAGAGGGCC
BV19	TCCTCTCACTGTGACATCGGCCCA
BV20	TGCCCCAGAATCTCTCAGCCTCCA
BV21	GGAGTAGACTCCACTCTCAAG
BV22	GATCCGGTCCACAAAGCTGG
BV23	ATTCTGAACTGAACATGAGCTCCT
BV24	GACATCCGCTCACCAGGCCTG
BJ1.1	TCTGGTGCCTTGTCCAAAGAAAGC
BJ1.2	CCTGTCCCCGAACCGAAGGTGTA
BJ1.3	CCAACTTCCCTCTCCAAAATATAT
BJ1.4	CTGGGTTCCACTGCCAAAAAACAG
BJ1.5	TCGAGTCCCATCACCAAAATGCTG
BJ1.6	CCTGGTCCCATTCCCAAAGTGGAG
BJ2.1	CCGTGTCCCTGGCCCGAAGAACTG
BJ2.2	CTAGAGCCTTCTCCAAAAAACAGC
BJ2.3	GGGTGCCTGGGCCAAAATACTGCG
BJ2.4	GGGTCCCGGCGCCGAAGTACTGAA
BJ2.5	CGCGTGCCTGGCCCGAAGTACTGG
BJ2.6	GCTGCCGGCCCCGAAAGTCAGGAC
BJ2.7	TGGTGCCCGGCCCGAAGTACTGCT

Using 2λ of this product as a template, run-off reactions were performed with a single internal fluorescent primer for each BJ tested. These products were then denatured in formamide and analyzed on an Applied Biosystem 3100 Prism using Gene-scan 2.0 software (Applied Biosystems, Foster City, CA, USA). Results are also reported as RSI (rate stimulation index = normalized peak area obtained from cells stimulated with antigen/normalized peak area of nonstimulated cells).

### Separation and immunoscopy analysis of CD45RA^+ ^and CD45RA^- ^T cells

To examine the expression of the CD45RA activation marker on T cells carrying the TCR rearrangements identified by TCR repertoire analysis, PBMCs from patient RE (who had been off therapy for 1 year) obtained during acute RA, were depleted of CD19^+ ^cells by magnetic MACS™ sorting (Miltenyi Biotec, Auburn, CA, USA), which was performed in accordance with the manufacturer's instructions. CD19-negatively sorted cells were enriched in CD45RA^+ ^and CD45RA^- ^cells by labelling them with an anti-CD45RA MACS™ beads, in accordance with the manufacturer's instructions. Enrichment for CD45RA and CD45RO was checked using a FACScan flow cytometer (Becton Dickinson). At least 5,000 cells of interest were acquired for each sample. A total of 2.5 × 10^6 ^CD45RA positively and negatively selected cells were co-cultured *in vitro *with 2 × 10^5 ^CD19^+ ^B cells as antigen-presenting cells, in the presence of 20 μg/ml collagen peptide. As a reference for the presence of antigen-driven expansions, 3.2 × 10^6 ^CD19-negatively selected cells were co-cultured *in vitro *with 2 × 10^5 ^CD19^+ ^cells, in the absence or presence of 20 μg/ml collagen peptide. Immunoscopy analysis for TCR cells in each sample was performed as described above.

### CDR3 sequencing

cDNAs were obtained from antigen-stimulated PBMCs or from cells obtained from the synovial fluid, as described above. Of each sample, 2 γ were submitted to an initial PCR, using the mentioned above with BV-specific forward primers and the common Cβ-specific reverse primer. A second nested PCR was then performed using 2 γ of the product of the former reaction as a template, with the same BV-specific primer and BJ-specific reverse primers. PCR fragments were then cloned using TOPO TA Cloning^® ^kit (Invitrogen, Carlsbad, CA, USA), in accordance with the manufacturer's instructions. Transformed *Escherichia coli *were grown in 5 ml LB medium supplemented with ampicillin, and plasmids were purified by Qiaprep Miniprep columns (Qiagen GmbH) and checked for the presence of the expected inserts by PCR amplification using BV-BJ paired primers. Samples that scored positive for the insert were sequenced using an M13 forward primer. DNA sequence was translated into protein sequence through the ExPASy Proteomics Server [26].

### CD4^+^CD25^+ ^cell depletion

Lymphocytes from peripheral blood were obtained as mononuclear cells by standard density gradient centrifugation of heparinized blood, as described previously [27]. All steps were performed in sterile phosphate-buffered saline containing 0.1% bovine serum albumin. Cell suspension was washed twice in cold phosphate-buffered saline-bovine serum albumin, resuspended at 10^6 ^cells/ml and used for subsequent studies. Because regulatory T (Treg) cells belong to this population, we investigated T-cell-specific proliferation in the presence and after depletion of the CD4^+^CD25^+ ^subset.

### Immunostaining

Optimal mAb concentrations were routinely determined for each mAb by titration. We used FITC-conjugated mAb to CD4 from Becton Dickinson Biosciences (San Jose, CA) and PE-conjugated mAb to CD25 from Miltenyi (Miltenyi Biotec, Auburn, CA, USA). Because there are no objective criteria by which to set the boundary between brightly and dimly stained CD25^+ ^cells (CD25^high ^and CD25^int^, respectively), all flow cytometric analyses were reviewed by one investigator (AB) who was blind to sample identity. Cells were measured by fluorescence-activated cell sorting immediately after staining using forward and side scatter signals to establish the lymphocyte gate and exclude unwanted material (nonviable cells, debris and cell clumps) from cell evaluation. Fluorescence signals were collected in log mode. A minimum of 5,000 cells of interest were acquired for each sample.

### Proliferation assay

As a measure of the inhibitory capacity of Treg cells contained in each sample, we measured the effect of CD4^+^CD25^+ ^depletion on the proliferative response of autologous T cells. The data were confirmed by adding back an excess of purified CD4^+^CD25^+ ^T cells to autologous T cells. Thus, circulating mononuclear cells were depleted immunomagnetically of CD25^high ^cells by microbeads directly coated with anti-CD25 mAb (Miltenyi Biotec GmbH, Friedrich-Ebert-Strasse 68 Bergisch Gladbach D- 51429, Germany). The amount of anti-CD25 was adjusted to target CD25^high ^cells preferentially, following a procedure that is standard in our laboratory. Concomitantly, the same mononuclear cell preparation was used to purify putative Treg cells. To this end, CD4^+ ^lymphocytes were first obtained as untouched cells by negative selection using the CD4^+ ^T cell Isolation Kit from Miltenyi, which contains CD8, CD11b, CD16, CD56, CD19 and CD36 mAbs, and microbeads directly coated with anti-CD25 mAb (Miltenyi) were then used to separate CD25^high ^cells, as described above. All other steps were performed following the manufacturer's instructions.

Depletion and purity were assessed by flow cytometry and found consistently to exceed 95% and 70% in CD4^+^CD25^+ ^(containing also Treg) depleted and Treg enriched preparations, respectively. The response of T cells to polyclonal activation was assessed using the intracellular covalent coupling dye carboxyfluorescein diacetate succinimidyl ester (CFSE, also referred to as CFDA-SE; Molecular Probes, Eugene, OR, USA) and TCR crosslinking as stimulus. The staining procedure was essentially as described previously [28]. Briefly, responder cells were aseptically loaded with 0.2 μmol/l CFSE, resuspended in RPMI 10% foetal bovine serum, and seeded (50,000 cells/well) in replicate wells in a standard 96-well culture plate for 5 days in the presence of plate-bound anti-CD3. Treg cells were added to autologous mononuclear cell preparation at a 1:1 responder/suppressor ratio. The proliferative response of T cells (hereafter referred to as 'proliferation index') can be quantified by ModFit™/Cell Proliferation Model™ software (Sigma, St Louis, MO, USA).

## Results

### Peripheral blood mononuclear cells that proliferate in response to huCollp261–273 exhibit a limited TCR usage at disease onset

We measured the proliferation of PBMCs obtained from a patient at the onset of RA, in response to graded amounts of peptides huCollp250–264, huCollp261–273 and huCollp289–303. According to the literature, a small but specific proliferation has been observed only in response to the peptide huCollp261–273.

**Figure 1:**
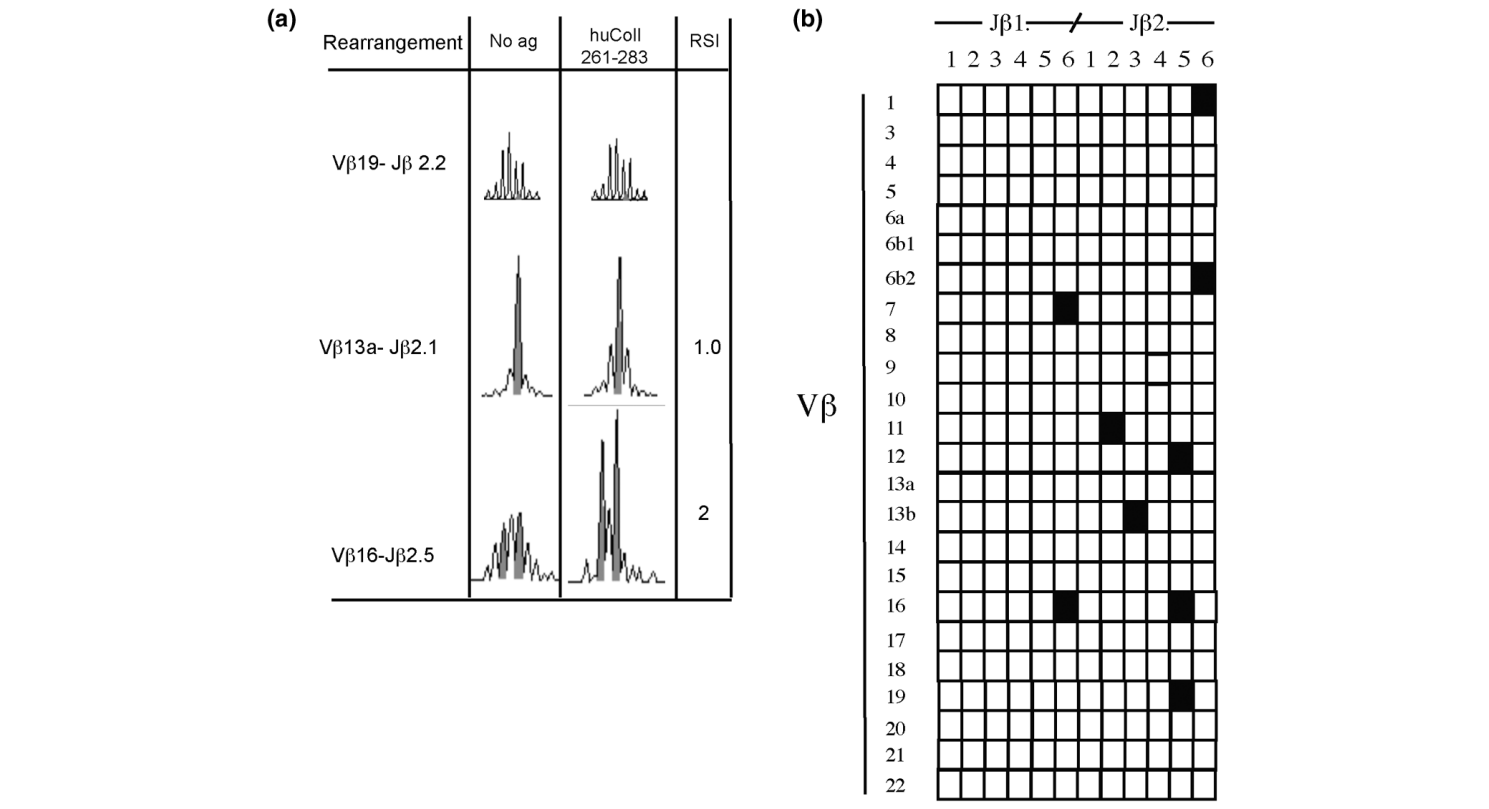
TCR repertoire usage in the immune response to huCollp263–271. PBMCs from patient OE were prepared as described in the Materials and methods section and cultured at 5 × 10^6 ^cells/ml in the presence or absence of 20 μg/ml huCollp263–271. Three days later cells were harvested and modified CDR3 β-chain spectratyping was performed, as described in Materials and methods. **(a) **Exemplificative BV-BJ CDR3 length spectra of T cells obtained for three rearrangements, in the absence and in the presence of antigenic peptide. The peaks interrupting the Gaussian distribution of CDR3 length (in an antigen-dependent or antigen-independent manner) are shaded in grey, and the RISs are shown. **(b) **Complete immunoscopy analysis of the immune response to huCollp263–271. Black squares indicate BV-BJ rearrangements, showing antigen-driven expansion (RSI ≥ 2) of one or more peaks. Rearrangement BV19-BJ2.5 exhibited an antigen-driven expansion of a peak of 99 bbases in length. In addition, expansion of two peaks of 96 and 105 bases in length was observed, with RSI 1.8. huCollp261–273, human collagen peptide 261–273; PBMC, peripheral blood mononuclear cell; RSI, rate stimulation index.

Therefore, PBMCs from the same blood sample stimulated with this peptide were used for immunoscopy analysis. In our analysis we studied a total of 288 spectra (encompassing approximately 85% of total BV-BJ rearrangements), each exhibiting 8 to 10 peaks. Each CDR3-β profile can be depicted as a function of the CDR3 length. Each peak represents three-base difference in the product of rearrangement, corresponding to one amino acid residue. According to immunoscopy analyses of other antigen-specific immune responses [29-32], after *in vitro *co-culture with huCollp261–273, BV-BJ CDR3-length fragment analysis of response to huCollp261–273 yields three distinct types of distribution of BV-BJ fragment length, as shown in Figure 1a. The great majority of rearrangements maintain the Gaussian distribution that is observed in the control (cultured in the absence of added peptide) sample, as for instances the reported BV19-BJ2.2. Such Gaussian distribution is perturbed in a second group of rearrangements, although not in an antigen-dependent manner (see, for instance, the spectra obtained for BV13a-BJ2.1; Figure 1a). In this case, the RSI of a candidate peak in the antigen-stimulated sample is usually below 1.5. In a third group of rearrangements (exemplified by rearrangement BV16-BJ2.5 in Figure 1a), however, the RSI between control and antigen-stimulated sample is 2 or greater.

According to our previous observations and to observations in the patients with early RA detailed in the following paragraph, perturbation of the Gaussian above this value identifies the group of T cells that depend on the presence of the specific peptide antigen to expand during *in vitro *culture, because the same perturbation is not elicited by stimulation with other antigen determinants [30-32]. As detailed in the Materials and methods section (above), selective spread of apoptotic cell death after antigen stimulation should contribute poorly to expansion of the few TCR rearrangements detected in these experimental conditions, because the number of annexin V positive T cells is similar between antigen-stimulated and control samples. Overall, this group of TCR rearrangements, which possibly associates with proliferating collagen-specific T cells, includes approximately 2% of total spectra examined.

The complete analysis of CDR3 length distribution is shown in Figure 1b. In addition to one expansion with an RSI of 2.5 (99 bases in length), rearrangement BV19-BJ2.5 exhibited expansion of two rearrangements with an RSI of 1.8 (96 and 105 bases in length). Spectra obtained for rearrangements BV11-BJ2.2 (135 and 138 bases) and BV16-BJ2.5 (83 and 86 bases) revealed the presence of two antigen-dependent expansions each. Collectively, a small number of rearrangements was found to expand with RSI of 2 or greater in an antigen-driven manner. Although we could not detect any obvious bias in BV usage, it appears that most cells expanding after antigen stimulation bear CDR3-β regions obtained through rearrangement of segments of the BJ2 family. This observation may imply that residues encoded by the BD2 gene segment play a role in the recognition of huCollp261–273.

### The repertoire detected by BV-BJ spectratyping in the patient comprises TCR rearrangements that are shared among DR4^+ ^patients with early RA and is specifically expanded by huCollp261–273

We collected PBMCs from five more patients at the onset of RA. We examined whether these patients also used any of the antigen-dependent rearrangements identified in the previous patients, at the onset or during the course of the disease. The results of our analysis are reported in Table 4, and show that a relatively large portion of the TCR repertoire used by the initially evaluated patients is also used by the other patients, thus representing a shared repertoire specific for huCollp261–273. Some of the shared rearrangements were used by more than one patient already at the onset of disease (such as BV11-BJ2.2 of 135 or 138 bases, BV13-BJ2.3 of 199 bases, or BV16-BJ2.5 of 83 or 89 bases). Others can be used at the onset or appear later during the disease course (for example, BV1-BJ2.6 of 134 bases, BV6b2-BJ2.6 of 215 or 218 bases, and BV16-BJ1.6 of 83 or 86 bases). However, a private repertoire for each patient is also available.

**Table 4:** BV-BJ CDR3 rearrangements expanded after huCollp261–273 stimulation in ERA (early rheumatoid arthritis) patients at onset and during follow up in peripheral blood and in synovial fluid

	Patient OE					Patient FA	Patient AV	Patient VR				Patient ST			Patient MS		
						
	Onset	Onset Syn^c^	Follow up 1	Follow up 2	Follow up 3	Onset	Onset	Onset	Onset syn^c^	Follow up 1	Follow up 2	Onset	Onset syn^c^	Follow up	Onset	Follow up 1	Follow up 2
DAS	6.7		3.5	3.2	6			5.2		2.6	3.7	6.2		1.6	6.2	1.6	1.6

BV-BJ																	

**1–2.6^a^**																	
134	**+**	**+**			**+**					**+**	**+**			**+**		**+**	
125						**+**										**+**	

Private^b^							131										

**6b2-2.6^a^**																	
209								**+**									**+**
215	**+**										**+**						
218											**+**	**+**					

Private^b^						203											

**7-1.6**	127	127			127						138						

**11-2.2^a^**																	
135	+										+				+		

138	+			+	+			+	+		+				+		+

Private^b^															131	131	

**13b-2.3^a^**																	
199	**+**							**+**	**+**		**+**	**+**	**+**		**+**		

193										**+**				**+**			**+**

201										**+**					**+**		

Private^b^																190	196

**19-2.5**	99																
	105																

**16-1.6^a^**																	
83	**+**				**+**		**+**	**+**			**+**						
86														**+**	**+**	**+**	

Private^b^														92	89	89	

**16-2.5^a^**																	
83	**+**						**+**										

89	**+**											**+**			**+**		

95											**+**				**+**		

Private^b^										77	87	80			97		97

	**11**	2	0	1	4	**2**	**3**	**4**	2	4	**7**	**4**	1	4	**10**	5	5

^a^The base length of each TCR rearrangement that is shared by two or more RA patients is listed in the first column, and its presence in PBMCs is indicated by '+'. ^b^TCR rearrangements that belong to private repertoires are indicated by the base length for each patient. ^c^Presence of an enrichment of the indicated rearrangement in T cells obtained from synovial fluid at onset of ERA. +, huColl261–273-driven detection of the indicated rearrangement; huCollp261–273, human collagen peptide 261–273; PBMC, peripheral blood mononuclear cell; RA, rheumatoid arthritis; TCR, T-cell receptor.

PBMCs from some of the patients studied also exhibited a small degree of proliferation after stimulation with the subdominant epitope huCollp289–303. In these patients, overall we identified 26 rearrangements expanded by stimulation with huCollp261–273 but not with huCollp289–303. Twenty-one rearrangements were expanded by huCollp289–303 but not by huCollp261–273. Only eight rearrangements were expanded by both peptides. At present we cannot distinguish whether cells carrying these rearrangements are heteroclitically activated by both epitopes or whether they expand as non-antigen-specific bystanders. These data confirm our observations in experimental models showing that BV-BJ spectratyping identifies, to a large extent, TCRs carried by antigen-specific T cells [30,31], as anticipated in the above paragraph.

### PBMCs of DR4^+ ^healthy individuals exhibit a shared TCR repertoire specific for huCollp261–273 distinct from the one used by patients with early RA

**Figure 2:**
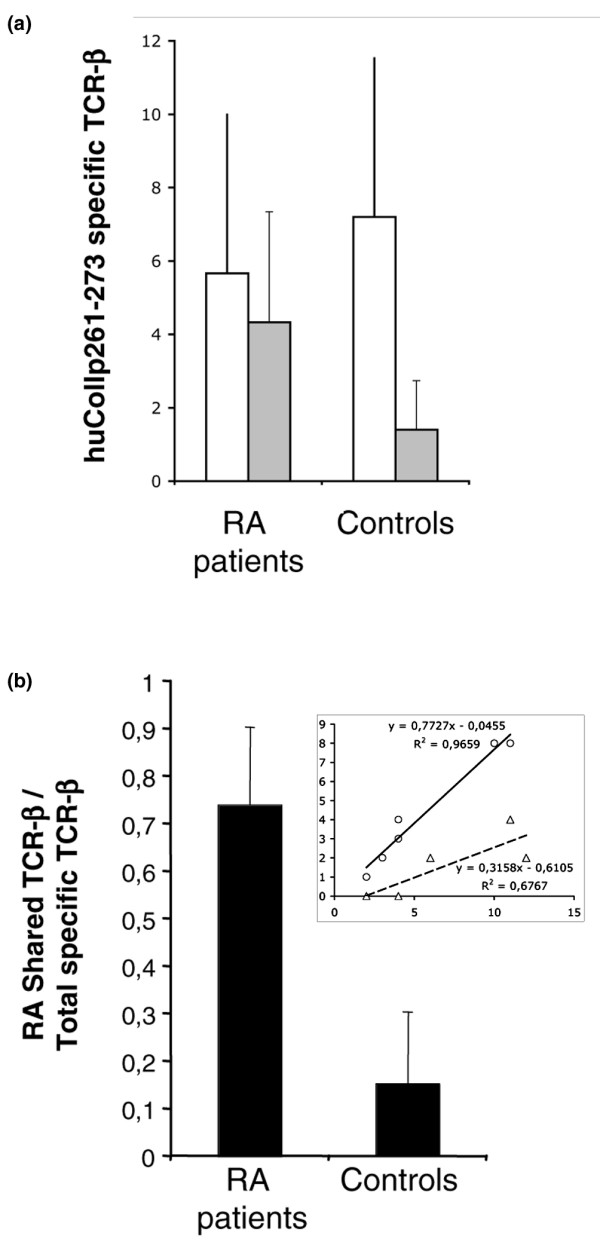
Ratio between shared and total TCR-β chains specific for huCollp263–271 discriminates RA patients from controls. **(a) **Average plus SD of the number of total (open bars) or shared (grey bars) TCR-β chains specific for huCollp263–271 in PBMCs of patients with early RA and control DR4^+ ^individuals. PBMCs from patients and control individuals were prepared as described in the Materials and methods section and cultured at 5 × 10^6 ^cells/ml in the presence or absence of 20 μg/ml huCollp263–271. Three days later, cells were harvested and modified CDR3 β-chain spectratyping for the rearrangements listed in Tables 3 and 4 was performed, as described in the Materials and methods section. **(b) **Average plus SD of the ratio between shared and total TCR-β chains specific for huCollp263–271 in PBMCs of patients with early RA and control DR4^+ ^individuals. Individual data are reported in the insert, in which the linear regression between total (x-axis) and shared (y-axis) TCR-β chains is shown for patients with early RA (circles) and control DR4^+ ^individuals (triangles). huCollp261–273, human collagen peptide 261–273; PBMC, peripheral blood mononuclear cell; RA, rheumatoid arthritis; SD, standard deviation; TCR, T-cell receptor.

We used the same approach to examine the huCollp261–273-specific repertoire in five healthy DR4^+ ^relatives of RA patients. The results of this analysis are detailed in Table 5 and are summarized in Figure 2. All tested control samples exhibited the presence of TCR expanding in response to stimulation of PBMCs with huCollp261–273. As shown in Figure 2, the total number of T cells expanding was no different among control individuals and RA patients (Figure 2a, open bars; *P *= 0.2). Usage of the previously defined 'shared' T-cell repertoire appears more frequent in RA patients versus control individuals (Figure 2a, dashed bars; *P *= 0.03). Consequently, the contributions of the shared TCRs to the total response exhibit clear differences between patients and control individuals (Figure 2b; *P *= 0.004). Patients with RA exhibit a ratio between the numbers of shared rearrangements to that of total rearrangements expanding of 0.5 or greater (and in most cases > 0.7); conversely, healthy control individuals have a value of 0.3 or less (in most cases < 0.2) for this ratio.

**Table 5:** BV-BJ CDR3 rearrangements expanded after huCollp261–273 stimulation in healthy control DR4^+ ^relatives of RA patients

BV-BJ^a^	MF (VR)^b^ 04–13 04–14	SR (ST)^b^ 04–04 01–04	ML (MS)^b^ 04–07	BI (BC)^b, c^ 04–04 01–04 04–09	GG (BC)^b, c^ 04–08
**1–2.6^a^**					
125,134					
other			**137^d^**	128, 131, **137^d^**	
**6b2-2.6^a^**					
209, 215, 218			215		
other		**212^(d)^**	**212^d^**		**212^d^**
**7-1.6**	142	127			139
**11-2.2^a^**				135, 138	
135, 138					
other		141	132		129
**13b-2.3^a^**					
193, 199, 201					193
other		196			
**19-2.5**	**101^d^**		95	104, **110^e^**	**101^d^, 110^e^**
**16-1.6^a^**			83		83, 86
83, 86					
other				89, 101	92
**16-2.5^a^**					
83, 89					83
other				77, **93^e^**, 96	**93^e^**
Ratio: RA shared TCRs/total specific TCRs	0/2	0/4	2/6	2/12	4/11

^a^BV-BJ rearrangements that are shared among RA patients are in bold. ^b^Initials of the name of the relative of the RA patient is given in brackets. Beneath each patient's initial the HLA DRB1 allele combination(s) are given, as established by PCR using sequence-specific oligonucleotides (see Materials and methods). ^c^BI and GG are both related to BC (see Table 6) and are third-degree relatives of each other. ^d^huCollp261–273 specific TCR-β rearrangements shared among healthy individuals. ^e^huCollp261–273 specific TCR-β rearrangements shared only by the two related individuals BI and GG. huCollp261–273, human collagen peptide 261–273; RA, rheumatoid arthritis; TCR, T-cell receptor.

Some of the studied TCR rearrangements specific for huCollp263–271, such as 1–2,6 (137 bases), 16b2-2,6 (212 bases) and 19-2,5 (101 bases), were shared among healthy control individuals but were not detected in RA patients. This observation suggests that T-cell repertoires of patients and healthy individuals are distinct, despite being specific for the same self-epitope.

### A small portion of the collagen-specific repertoire spontaneously accumulates in inflammatory synovial fluid

**Figure 3:**
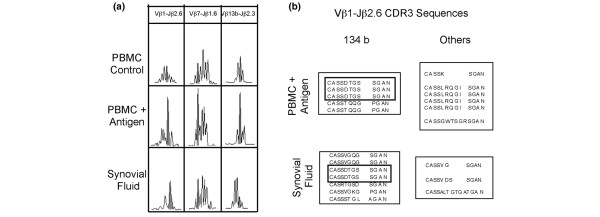
Some T cells that expand in response to huCollp263–271 are spontaneously enriched in synovial fluid. **(a) **BV-BJ spectra of three rearrangements were obtained from PBMCs of patient OE cultured in the absence (control) or presence of huCollp263–271, or from cells harvested from inflamed knee joint. **(b) **CDR3 sequences of TCRs carrying BV1-BJ2.6 obtained from collagen peptide-stimulated PBMCs or inflammatory synovial fluid from patient OE. A box indicates that CDR3 sequences of the expected length that are present in multiple copies in both samples. huCollp261–273, human collagen peptide 261–273; PBMC, peripheral blood mononuclear cell; TCR, T-cell receptor.

We examined whether any of the TCR CDR3-β regions carried by T cells proliferating in response to huCollp261–273 were enriched spontaneously in synovial fluid during the acute disease. We collected synovial fluid from three patient (OE, VR and ST) and isolated cells after centrifugation. mRNA and cDNA were prepared from these cells, without prior antigen stimulation, as described in the Materials and methods section (above). Finally, we conducted the immunoscopy analysis for the nine BV-BJ rearrangements that had exhibited alteration in Gaussian distribution associated with huCollp261–273-specific proliferation. The results are reported in Figure 3 and Table 4. Figure 3 presents data from patient OE. We observed that two spectra (namely BV1-BJ2.6 and BV7-BJ1.6) obtained from analysis of cells of the inflammatory synovial fluid exhibited spontaneous expansion of the same peaks (134 and 127 bases, respectively) that expanded in PBMCs after antigen stimulation. On the contrary, all of the other spectra behaved as was shown for BV13-BJ2.3, in which the spectrum obtained from the synovial fluid sample overlaps with that obtained from the unstimulated PBMCs.

To confirm that the BV1-BJ2.6 (134 bases) TCR detected in the synovia was derived from the same T cells that expand in response to huCollp261–273, we cloned and sequenced this rearrangement from huCollp261–273-stimulated PBMCs and from synovial cells. The results (Figure 3b) indicate that one CDR3 sequence (CASS DTGS SGAN) was obtained from both samples, in multiple copies. This CDR3 exhibits the expected length for the huCollp261–273-specific CDR3. *Vice versa*, large variability in CDR3 sequences was observed among shorter or longer CDR3s between the two samples. These data suggest that CASS DTGS SGAN may be a sequence characterizing this huCollp261–273-specific CDR3-β region.

In Table 4 we show that only a fraction of the collagen-specific TCRs detected in the blood appeared enriched spontaneously in synovial fluid in all tested samples. Although four out of five rearrangements detected belong to the group of the shared rearrangements, T cells enriched in the synovial fluid were different in each patient.

### huCollp261–273-specific repertoire is downmodulated in peripheral blood during the moderate disease activity/remission of disease induced by therapy

The antigen-driven expansion of huCollp261–273-specific rearrangements was examined in four patients (OE, VR, ST and MS) after a consistent improvement in disease activity had been achieved. The data are presented in Table 4.

A downregulation of the repertoire responsive to huCollp261–273, detected at the onset of the disease, generally occurred in most RA patients after clinical remission. In some cases, elevated levels of new specific TCRs were also observed (Table 4).

The behaviour of T cells carrying the rearrangement BV11-BJ2.2 of 138 bases in length (hereafter referred to as BV11^+ ^cells) is particularly noteworthy. The presence of huCollp261–273-specific T cells carrying this rearrangement was detected in three out of the six patients with early RA. After disease remission, BV11^+ ^cells were no longer detected. They were again detected in two patients, OE and MS, when disease activity was still low. Intriguingly, a clinical relapse of the disease was observed at the following clinical control 3 and 1 months later, respectively. Also, the third patient, VR, once again exhibited usage of this rearrangement in coincidence with disease relapse. These observations suggest that BV11^+ ^T cells are related to disease activity. We therefore examined the presence of BV11^+ ^T cells in six more DR4^+ ^RA patients during stable remission (three patients) or activity (three patients) of the disease (Table 6). BV11^+ ^T cells were detected in two out of the three samples taken during acute bouts of RA, and in none of the patients during remission.

**Table 6:** Detection of BV11^+ ^T cells specific for huCollp261–273 is associated with disease activity

Disease status	Patient	Disease stage	DAS	BV11+ cellsa
Active	OE	Onset	6.7	**+**
	FA	Onset	6.0	-
	AV	Onset	6.2	-
	VR	Onset	5.2	**+**
	ST	Onset	6.2	-
	MS	Onset	6.2	**+**
	OE	Relapse	6.0	**+**
	VR	Relapse	3.7	**+**
	VG	Relapse	3.7	-
	BC	Relapse	3.7	**+**
	RE	Relapse	4.8	**+**

Moderate disease activity/remission	OE		3.5	-
	VR		2.6	-
	ST		1.6	-
	MS		1.6	-
	OE		3.2	+^b^
	MS		1.6	+^b^
	VGC		0.46	-
	MP		1.22	-
	LM		0.9	-

^a^+ presence or – absence of the huColl261–273 driven expansion of the BV11-BJ2.2 (138 bases) peak in peripheral blood mononuclear cells. ^b^Patient relapsed at the following DAS control. DAS, Disease Activity Score; huCollp261–273, human collagen peptide 261–273.

It is possible to detect BV11^+ ^cells in PBMCs before disease relapse. However, acute relapse of disease is also associated with detection in the PBMCs of the same cells that were spontaneously enriched in the synovial fluid at the onset (Table 4).

Taken together, these observations further strengthen the hypothesis that the identified rearrangements belong to disease-related TCRs. In addition, they suggest that improving the disease by treatment is mirrored by modulation of the ability of T cells to respond to collagen peptides.

### CDR3-β of BV11-BJ2.2 of 138 bases and BV13-BJ2.3 of 199 bases present overlapping motifs in their sequences among different patients

In both human and experimental models, shared TCR-β chains display similar CDR3 sequences. To test the hypothesis that at least the two most frequent TCR rearrangements specific for huCollp261–273 are actually shared among early RA patients, we sequenced the BV11-BJ2.2 chains from huCollp261-stimulated cells of patients OE, VR, MS and BC at the time of clinical relapse, and the BV13-BJ2.3 chains from huCollp261–273-stimulated cells of patients OE, VR, ST and MS at the onset of disease. Sequences obtained for BV11-BJ2.2 of 138 bases and BV13-BJ2.3 of 199 bases are reported in Table 7.

**Table 7:** Amino acid and cDNA sequences of the shared BV11-BJ2.2 (138 bases b) and BV13b-BJ2.3 (199 bases b) TCR CDR3 region in ERA (early rheumatoid arthritis) patients

Region	Patient	Aminoacid sequences^a^	Corresponding cDNA sequence^b^
BV11-BJ2.2 (138b)	OE	C A S **S E S R Y** T	TGTGCCAGCAGTGAATCCCGTTATACCGG
		C A S **R G Q P N** T	TGTGCCAGCCGCGGACAGCCAAACACCGG
		C A S **R G Q P N** T	
	
	VR	C A S **S E P R N** T	TGTGCCAGCAGTGAA**C**C**TAGGAAC**ACCGG
		C A S **S E P R N** T	
		C A S **S E P R N** T	
		C A S **S E P R N** T	
		C A S **S E P R N** T	
		C A S S A V R N T	
		C A S S P A G N T	
		C A S T P S G G T	
		C A S S E L A D T	
		C A S S G R T S T	
	
	MS	C A S **R G Q P N** T	TGTGCCAGCCGCGGACAGCCAAACACCGG
		C A S **R G Q P N** T	
		C A S **R G Q P N** T	
		C A S **R G Q P N** T	
		C A S **R G Q P N** T	
		C A S **R G Q P N** T	
		C A S **R G Q P N** T	
		C A S **R G Q P N** T	
		C A S **R G Q P N** T	
		C A S **S E P R N** T	
		C A S S E N W N T	TGTGCCAGCAGTGAA**C**C**TAGGAAC**ACCGG
		C A S T R D G H T	
		C A S S E N W N T	
		C A S S K S G L S	
	
	BC	C A S** S E S F N** T	TGTGCCAGCAGTGAATC**GTTTAAC**ACCGG
		C A S **R G Q P N** T	
		C A S **R G Q P N** T	TGTGCCAGCCGCGGACAGCCAAACACCGG
		C A R Q D G D H T	
		C A S S G L A G A	

BV13bBJ2.3	OE	C A S S **E A S G S T** D	TGTGCCAGCAGTGAGGCTAGCGGGAGCACAGATA
(199b)		C A S S A V P G Q R D	
		C A S S L S G T A P D	
	
	VR	C A S S **L F S G D T** D	TGTGCCAGCAGT**TTATTTA**G**C**GG**G**G**AT**ACAGATA
		C A S S **L F S G D T** D	
		C A S S L H P G P T D	
		C A S S T Q A A S T D	
		C A S S L H P G P T D	
	
	ST	C A S S **R T V G D T** D	TGTGCCAGCAG**CCGGACTGTA**GGG**GAC**ACAGATA
		C A S S **R T V G D T** D	
		C A S S **R T V G D T** D	
		C A S S D V G T G R D	
		C A S S D V G T G R D	
		C A S S D V G T G R D	
	
	MS	C A S S **L S G G GT** D	TGTGCCAGCAGT**CTCAGCG**G**G**GG**AGGG**ACAGATA
		C A S S D P D T S T D	
		C A S S E S G V A T D	
		C A S S V L A G KA D	
		C A S R V T G G P E L	

^a^Sequences corresponding to the shared motifs are presented in bold, underlined text. ^b^Bases different from those found in the index patient OE are presented in bold. TCR, T-cell receptor.

We sequenced 87 BV11-BJ2.2 chains (18 from patient OE, 23 from VR, 26 from MS and 20 from BC). As shown in Table 7, each sample displayed multiple sequences of the expected 138-base length of one (VR) or both (OE, MS and BC) sequences C A S R G Q P N T G E L and C A S S E P^S ^R^F ^N^Y ^T G E L.

The corresponding cDNA sequences were equal among all of the patients for the C A S R G Q P N T G E L motif. Despite the fact that often the same amino acid residue within a public CDR3 sequence is encoded by distinct triplets, others have reported that common TCR-β chains found in the synovia of two RA patients were encoded by the same nucleotide sequence [21]. In our case, PCR amplifications and cloning for sequencing for each patient were performed in distinct experiments over several months, and by different operators, and this procedure should have minimized the possibility that contamination occurred.

A few differences were found in cDNAs encoding the C A S S E P^S ^R^F ^N^Y ^T G E L motif, which accounted for the resulting differences in the amino acid sequences. The final tract of the germline sequence for BV11 is TGTGCCAGCAGTGAATA and the first 15 nucleotides of this stretch encode the amino acid sequence CASSE. In two of our samples (OE and BC) the germline T was also maintained, whereas it appeared to be deleted and substituted during the V-DJ rearrangement in both VR and MS. The frequency of CASSE in the BV11-JB2.2 rearrangement was roughly 25% in each group of length (132 bases to 144 bases). However, we found the suggested core motif C A S S E P^S ^R^F ^in nine out of 10 sequences starting with CASSE of 138 bases in length, and in only in one out of 21 sequences starting with CASSE of 141 bases and 144 bases in length (not shown). Thus, this motif may also be related to response to huCollp261–273, and the two motives RGQPNT and SEPRNT together may represent the shared rearrangements for BV11-BJ2.2 of 138 bases. We did not find any obvious mechanism (such as, for instance, linkage to the other DRB1 allele) by which some patients use only one and others use both CDR3 rearrangements.

A total of 81 BV13b-BJ2.3 TCRs were sequenced (14 from patient OE, 13 from VR, 28 from ST and 26 from MS). In this case, we found no obviously similar sequences, although we found at least one sequence of the expected length (199 bases) per each patient exhibiting the following motif: C A S S X X S^V^_G _G D^S^_G _T D T Q. This motif is characterized by the presence of a G and a T spaced by one amino acid residue; this amino acid is either S or D in patients OE, ST and VR. Both S and D have a polar side chain, although that of D is negatively charged and that of S is not. In all of the samples, the amino acid preceding the conserved G has a short side chain (S in patients OE and VR, V in patient ST, and G in patient MS), although S is a polar amino acid whereas G and V are not. If these TCR-β chains were specific for huColl261–273/HLA DR4 complexes, then the data suggest that G and T possibly play a dominant role in establishing direct contact with the antigen, whereas the flanking residues may be more relevant in determining the appropriate tertiary structure [30]. The corresponding cDNA sequences exhibited differences in their composition that are in part also accounted for by codon degeneration.

Overall, we found no obvious similarity between the sequences we obtained for collagen-specific expansions (including that of the BV1-BJ2.6^+ ^cells homing to the synovia) and those that were described in the synovia of a large collection of RA patients [21]. This difference may be due to the fact that the cohort of patients we studied was composed exclusively of DRB1 04^+ ^patients, and that we focused only on huColl261–273-specific cells.

### TCRs that expand in antigen-driven manner belong mostly to CD45RA^- ^T cells

CD45 isoforms and CD62L in parallel describe distinct subsets of T cells. Effector and effector/memory T cells are CD62L^low^, CD45RA^- ^and CD45R0^+^, whereas naïve and central/memory T cells are CD62L^high^, CD45RA^+ ^and CD45R0^- ^[32]. We used this discrete distribution of surface markers to establish the population to which T cells identified by immunoscopy as specific for huCollp261–273 belong.

PBMCs were obtained from patient RE, who had a DAS 4.8. A portion of the cells was separated into CD19^+ ^and CD19^- ^populations by magnetic sorting. CD19^- ^cells were then subjected to a further step of magnetic sorting for the enrichment of CD45RA^+ ^and CD45RA^- ^cells. Details of the expression of CD45 isoforms on the enriched populations are reported in Table 8.

**Table 8:** Distribution of huColl261–273 specific T cells between CD45RA-enriched and CD45RA-depleted cells

Sample composition	PBMC	Total CD19^-^/CD19^+^	CD19^- ^CD45RA enriched/CD19^+^	CD19^- ^CD45RA depleted/CD19^+^
CD45 isoform expression (% of CD3^+ ^cells)	CD45RA^+ ^32%		CD45RA^+^: 74% CD45RO^+^: 4.2%	CD45RA^+^: 0% CD45RO^+^: 54%
Rearrangement				
BV11-BJ2.2 (138 bases)	**+**	**+**	**-**	**+**
BV13-BJ2.3 (201 bases)	**+**	**+**	**+**	**-**
BV16-BJ1.6 (86 bases)	**+**	**+**	**-**	**+**
BV16-BJ2.5 (81 bases)	**+**	**+**	**-**	**+**

Shown is the presence (+) or absence (-) of the huColl261–273 driven expansion of the indicated rearrangements. huCollp261–273, human collagen peptide 261–273.

The four populations (PBMCs, total CD19^-^, CD45RA-enriched CD19^- ^[74% CD45RA^+^], CD45RA-depleted CD19^- ^cells [0% CD45RA^+ ^cells]) were cultured in the absence or presence of peptide antigen and of CD19^+ ^cells (as antigen-presenting cells) for those populations that had been depleted of CD19^+ ^cells. After 3 days, cells were harvested and subjected to immunoscopy analysis for the BV-BJ rearrangements under study. The results are reported in Table 8. PBMCs from patient RE exhibited usage of four TCR rearrangements, three of which (BV11-BJ2.2 [138 bases], BV13-BJ2.3 [201 bases] and BV16-BJ1.6 [86 bases]) were shared with other RA patients and one (BV16-BJ2.5 [81 bases]) was exhibited solely by this individual patient. All of these rearrangements were once again found (as expected) in the control sample that was reconstituted by adding 3.2 × 10^6 ^CD19^- ^cells and 2 × 10^5 ^CD19^+ ^cells. When we checked for the presence of each of the rearrangements in the CD45RA-enriched or -depleted populations, we found that three rearrangements (BV11-BJ2.2 [138 bases], BV16-BJ1.6 [86 bases] and BV16-BJ2.5 [81 bases]) were enriched in the CD45RA^- ^population, whereas cells carrying the BV13-BJ2.3 201-base rearrangement co-eluted with CD45RA^+ ^cells.

To confirm the enrichment of BV11^+ ^cells in the CD45RA-depleted population, we sequenced BV11-BJ2.2 CDR3 in the CDR45RA-enriched and -depleted populations. Twelve sequences were obtained from the CD45RA-enriched population. Only one was of the expected 138 bases in length and exhibited the following amino acid sequence: C A S S E S G L S G E L. Six sequences were obtained from the CD45RA-deleted sample. Two of them were of the expected 138 bases in length and both exhibited the sequence C A S R G Q P N T G E L, corresponding to one of the shared CDR3 of BV11^+ ^public cells. Thus, CDR3 sequencing confirms that BV11^+ ^cells detected in the peripheral blood of RA patients during acute disease display an effector/effector memory phenotype. Similarly, most of the TCRs that we identified as associated with the response to huColl261–273 during acute bouts of disease display this effector/effector memory phenotype. At present we cannot establish whether cells that co-elute with CD45RA^+ ^cells belong to the central memory or to a naïve cell population. However, their ability to proliferate promptly in the presence of antigen without need for exogenously added cytokines rather resembles what is expected of central memory cells [32].

### Depletion of CD4^+ ^CD25^+ ^cells from PBMCs does not restore proliferation *in vitro *of the original TCR repertoire

Several mechanisms can play a role in the abrogation of the antigen-driven proliferation of huCollp261–273-specific T cells. They include depletion from peripheral blood of T cells because of clonal exhaustion [33] or complement-mediated lysis [35,36] (although this mechanism does not appear to be relevant in the therapy based on tumour necrosis factor [TNF]-α blockers), as well as restoration of regulatory circuits. It has been reported in fact that development of RA is linked to a decrease in suppressor activity of CD4^+^CD25^+ ^suppressor cells [37]. It has also been observed that treatment with the TNF-α blocker raises the number of these cells that can thus downmodulate self-reactivity [38,39]. We tested the hypothesis that failure of the original repertoire to expand *in vitro *after stimulation with huCollp261–273 was due to the suppressive activity of CD4^+ ^CD25^+ ^cells. We therefore compared the antigen-driven expansion of the nine rearrangements in total PBMCs and in PBMCs that had been depleted of CD4^+ ^CD25^+ ^cells by immunoaffinity.

**Figure 4:**
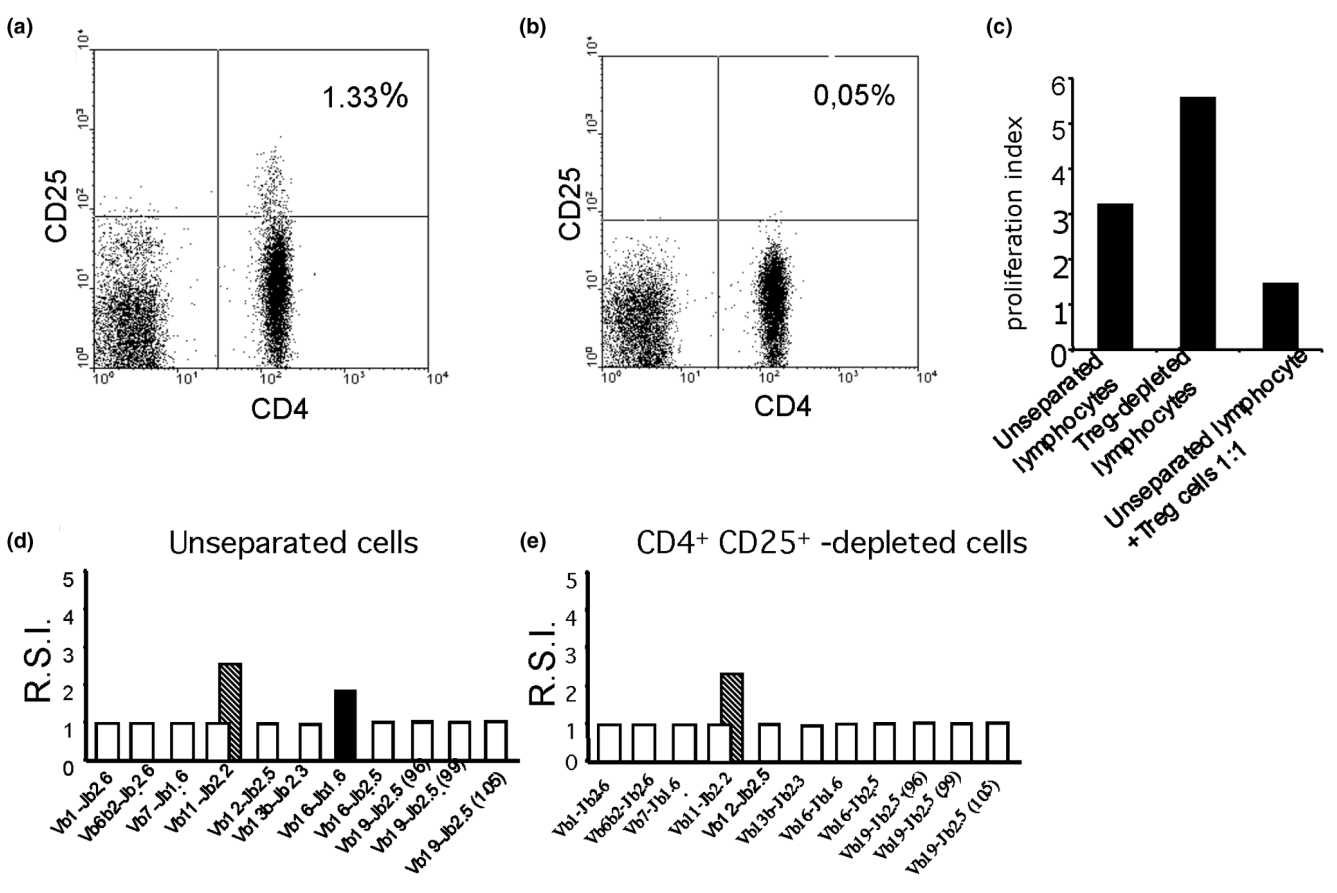
CD4^+^CD25^+ ^cell depletion from PBMCs does not restore proliferation *in vitro *of the original TCR repertoire. **(a, b) **Dual colour dot plot displaying the co-expression of CD4 and CD25 in the lymphocyte population. The regions used to measure the proportion of CD4^+^CD25^high ^(Treg cells) are shown. (Panel a) Treg cells are 1.33% and 2.9% in the total lymphocyte population and within the CD4^+ ^subset, respectively. (Panel b) Treg cells are efficiently removed (0.05% and 1.1% in the total lymphocyte population and within the CD4^+ ^subset, respectively) after depletion with anti-CD25 immunomagnetic microbeads. **(c) **Effect of Treg cell removal and of excess Treg cells on T-cell proliferative response. Bars show the proliferation index measured by ModFit™/Cell Proliferation Model™ software, as detailed in the Materials and methods section. **(d) **RSI of the TCR repertoire involved in the response to huCollp261–273 when examined in the unseparated PBMCs (see panel a). Dashed bars indicate the RSI of the BV11-BJ2.2 (138 bases) rearrangement. **(e) **RSI of the TCR repertoire involved in the response to huCollp261–273 when examined in the CD4^+^CD25^+^-depleted PBMC. huCollp261–273, human collagen peptide 261–273; PBMC, peripheral blood mononuclear cell; RSI, rate stimulation index; TCR, T-cell receptor.

Figure 4 shows CD4^+^CD25^+ ^cell content in the lymphocyte population (panel a) and efficiency of CD4^+^CD25^+ ^cell depletion (panel b) in the second sample obtained from patient OE during remission. The enhancing effect on lymphocyte proliferative response to TCR crosslinking after CD4^+ ^CD25^+ ^T-cell depletion and the blocking effect of an excess of this T-cell subset is detailed in Figure 4c. These data show that functionally active Treg cells were present in the PBMCs of patients. However, the immunoscopy analysis reported in Figure 4 panels d and e revealed that depletion of CD4^+^CD25^+ ^cells did not restore the proliferative ability of any of the rearrangements that displayed antigen-driven expansion in the first analysis.

In addition, we also observed that T cells carrying the BV16-BJ1.6 (83 bases) rearrangement (that belong to the 'original' repertoire) were lost in the fraction that is depleted of CD4^+^CD25^+ ^cells. This observation suggests that a part of the collagen-specific repertoire may play a regulatory role.

## Discussion

In this report we used modified BV-BJ immunoscopy to analyze a part of the TCR repertoire involved in the early proliferative response to huCollp261–273 in 12 DRβ 1 04^+ ^RA patients and 5 DRβ 1 04^+ ^healthy relatives of RA patients. The CDR3 BV-BJ spectratyping (that is about 10-fold more sensitive than the classical BV-BC spectratyping) has shown to be able to produce a detailed picture of immune responses [29,40]. This technique needs a single round of proliferation '*in vitro*' to highlight specific T cells in lymph nodes or peripheral blood, and allows the identification of a given TCR in target organs also [29,31,32]. In mouse this technique has allowed the dissection of antigen-specific T-cell repertoires (for examples [29,31,32]), showing that T-cell responses are not spread on the entire available repertoire of CDR3-β regions. Rather, a limited number of rearrangements is responsible for the entire response. Usage of these rearrangements can be individual (the private repertoire) or shared among a reasonable percentage of individuals (semi-private and public repertoires). Public TCRs were identified in response to influenza matrix protein, cytomegalovirus, Epstein-Barr virus, HIV and simian immunodeficinecy virus in humans [41].

In the index case, in which complete BV-BJ spectratyping was performed, we observed that the patient used a limited repertoire of TCR at onset of the disease. It was possible to define a group of TCRs specific for huCollp261–273 shared among RA DRB1 04^+ ^patients and distinct from the repertoire that is shared among healthy control individuals. A part of the collagen-specific repertoire was spontaneously enriched in the synovial fluid obtained from the inflamed joint. In addition, a large part of the repertoire detectable by immunoscopy during acute presentation of RA appears to belong to the effector CD45RA^- ^population. These two observations together support the hypothesis that collagen-specific T cells were direct bystanders of the acute presentation of the disease. Therapy that reduced disease activity also reduced the proliferation of this repertoire in response to huCollp261–273, and few new rearrangements are recruited in the original collagen-specific TCR repertoire during the course of the disease. Depletion of CD4^+^CD25^+ ^cells did not restore the antigen-driven expansion of T cells carrying the studied rearrangements, suggesting that suppressor activity induced by therapy is apparently not involved in the failure to proliferate of the studied cells. It must be recognized that not all Tregs are depleted by depleting CD4^+^CD25^+ ^cells. Nevertheless, our results show that collagen-driven T-cell expansion does not depend strictly on their function.

In the present study we found that the TCR repertoire used by this patient in the early phases of RA appears fairly limited. In addition, only a few of the available huCollp261–273-specific T cells actually homed to the synovia. Disease activity remained low also when the BV11^+ ^self-reactive repertoire reappeared and once again became detectable, whereas DAS was once again high only when also those CDR3s that identify T cells homing to the synovia at the onset of disease were again detected in PBMCs. Therefore, it may be suggested that the acute event relies on those CDR3s that belong to T cells that can to home to the synovia. Thus, a therapeutic intervention may focus on these T cells without the need to deplete the entire self-reactive repertoire to achieve clinically relevant results. In addition, the presence of the BV11^+ ^cells in PBMCs may prove helpful in managing RA patients, if studies in a large cohort can confirm that T cells belonging to this repertoire re-emerge ahead of disease reactivation.

Healthy relatives of RA patients show usage of a special set of collagen-specific shared TCRs, distinct from those found in patients. Further studies will be needed to establish whether these collagen-specific T cells rather protect from disease. At least two potential mechanisms may explain this modification of the self-reactive repertoire observed. In the first possible mechanism, RA-prone patients may be predisposed to rearrange and allow the maturation in the thymus of T cells carrying TCRs that are different from those produced in the DR4^+ ^patients, who will not develop disease. Such a predisposition will result in T cells having a higher avidity for self-antigens and/or a greater propensity of these cells to polarize toward pathogenic phenotypes such as T-helper-1 or T-helper-17. A second hypothesis is that the encounter with an environmental crossreactive antigen causes a shift of the collagen-specific TCR repertoire from that related to healthy status to one involved in disease determination [32].

The observation that the TCR repertoire mostly involved in disease determination remains fairly stable during disease conveys interesting theoretical suggestions. Despite small clonotype enlargement, we observed that it is in the majority of the cases the initial repertoire that can be found in blood also at later time points. Such a failure to modify the TCR repertoire of self-reactive cells can be the result of two mechanisms. One possibility is that central tolerance may reduce the autoreactive repertoire. Alternatively, memory T cells persisting in lymph nodes can prevent priming of naïve T cells by competition for the antigen-presenting cell [42-44]. In any case, early blockade of the disease might prevent the late spread of TCR usage.

It has been suggested that CD4^+^CD25^+ ^cells downregulate immune responses acting via cell-cell contact or cytokine secretion [45]. Their decrease has been associated with development of self-reactive diseases (for example [46]). In RA, however, it has been reported that the number of CD4^+^CD25^+ ^cells in peripheral blood is comparable to that in normal individuals, and that they accumulate in inflamed joints [47,48], where they also suppress secretion of protective cytokines [48]. Treatment with TNF-α blocker in RA patients improves the suppressive activity of these cells [38], possibly preventing their apoptosis [49], which can thus downmodulate self-reactivity [37]. However, failure of collagen-specific T cells to proliferate in response to peptide antigen did not appear in this case to rely on CD4^+^CD25^+ ^cells (which contain the Treg cells), even though the CD4^+^CD25^+ ^T cells were demonstrated to be functionally active. It can be hypothesized that other mechanisms may be employed to regulate the presence of self-reactive T cells during disease, such as activation-induced cell death and presence of interleukin-10 or transforming growth factor-β producing cells. However, our data do not permit discrimination among these or other mechanisms.

Further studies in early RA patients could provide more strong data to support the hypothesis that the CDR3 sequences we observed in this case do represent a common repertoire of the TCR among T cells that are self-reactive to huCollp261–273. Were this true, then new strategies could be envisioned with diagnostic and therapeutic applications.

## Conclusion

The huCollp261–273-specific TCR repertoire in peripheral blood and synovial fluid is restricted to a limited number of TCR-β rearrangements. The majority of the repertoire is shared between patients with early RA and it is modulated by therapy. Monitoring the self-reactive TCR repertoire in peripheral blood may allow prediction of disease relapse, providing a powerful tool for patient management.

## Abbreviations

CDR3: third complementarity-determining region; CFSE: carboxyfluorescein diacetate succinimidyl ester; DAS: Disease Activity Score; HLA: human leucocyte antigen; huCollp261–273: human collagen peptide 261–273; mAb: monoclonal antibody; PBMC: peripheral blood mononuclear cell; PCR: polymerase chain reaction; RA: rheumatoid arthritis; RSI: rate stimulation index; TCR: T-cell receptor; TNF: tumour necrosis factor; Treg: regulatory T (cell).

## Competing interests

The authors declare that they have obtained a national patent RM2007A000429 and an international PCT deposited on 6 August 2008: PCT/IB2008/053152National Patent.

## Authors' contributions

FR has made substantial contributions to the conception and design of the study, and analysis and interpretation of the data. He drafted the manuscript and gave final approval of the version to be published. RP, MDS, CN, GDS, AF and AB conducted the research. MO and DA contributed analytical tools. GF made substantial contributions to the conception and design of the study, and analysis and interpretation of the data. He drafted the manuscript and gave final approval of the version to be published.
